# Expanding the Scope of Mentoring for Psychiatry Trainees in Northern Ireland

**DOI:** 10.1192/bjo.2022.121

**Published:** 2022-06-20

**Authors:** Sarah Davidson, Meta McGee, Julie Anderson, Stephen Moore

**Affiliations:** 1 Northern Health and Social Care Trust, Antrim, United Kingdom; 2 Southern Health and Social Care Trust, Craigavon, United Kingdom; 3 Northern Ireland Medical and Dental Training Agency, Belfast, United Kingdom; 4 South Eastern Health and Social Care Trust, Belfast, United Kingdom

## Abstract

**Aims:**

The Northern Ireland psychiatry mentoring scheme, in which higher trainees mentor core trainee year 1 (CT1) doctors, has been running for four years. In this year's scheme, implemented in August 2021, we have expanded the scope of the scheme and implemented an online platform to match and connect mentors and mentees. Our aim was to gather baseline data regarding the experiences of mentors and mentees and to capture information regarding the content of mentoring meetings and attitudes towards format of meetings.

**Methods:**

Higher psychiatry trainees were invited to sign up as mentors through the Northern Ireland Medical and Dental Training Agency (NIMDTA) and Royal College of Psychiatry Northern Ireland (RCPsych NI) mailing lists. Mentors were obliged to complete a theoretical module on training before meeting their mentees. Core trainees in the first and second year of training were asked to opt-out of the scheme if they preferred not to be involved. CT3 trainees were offered the opportunity to opt-in to the scheme. There were a total of 16 mentors and 22 mentees at the outset. The NIMDTA Professional Support Unit provided an online platform, Mentornet, which allowed mentors and mentees to complete a profile, for mentees to rank their preferences for mentor, and to facilitate meetings. One of the authors (M.M.) presented the developments in the scheme to a nationwide audience in the RCPsych webinar on mentoring.

**Results:**

Six mentors and two mentees responded to the call to complete a baseline online questionnaire. 83% of mentors responded that they had found their role enjoyable and rewarding, whilst 67% of mentors indicated that their role had helped them develop in other skill areas. Both mentees responded that they had found the scheme beneficial and would recommend participation to other trainees.

**Conclusion:**

Mentorship is a valuable opportunity for senior psychiatry trainees to facilitate the professional development of junior trainees and to pass on their experience. This is the first year that all core trainees have been invited to participate and that a new web platform has been used to facilitate meetings. Baseline feedback response numbers have been limited although the responses were universally positive. We intend to obtain further feedback at the end of this year in order to devise quality improvement measures for the 2022/2023 cohort.

